# From guidelines to global impact: updates to address disparities in hereditary angioedema care

**DOI:** 10.1186/s13223-025-01008-8

**Published:** 2025-12-16

**Authors:** Ankur Kumar Jindal, Philip H. Li

**Affiliations:** 1 Division of Paediatric Clinical Immunology and Rheumatology, Department of Paediatrics, Manipal Hospitals, Bengaluru, India; 2 Division of Rheumatology and Clinical Immunology, Department of Medicine, Queen Mary Hospital, University of Hong Kong, Hong Kong, China; 3 Division of Rheumatology and Clinical Immunology, Department of Medicine, University of Hong Kong- Shenzhen Hospital, Shenzhen, China

## Abstract

Hereditary angioedema (HAE) is a rare, potentially life-threatening genetic disorder characterized by recurrent subcutaneous and/or submucosal edema, most commonly caused by deficiency in C1-esterase inhibitor. There has been a paradigm shift in treatment goals—from merely managing acute attacks to improving or normalizing quality of life, thanks to the availability and accessibility of modern, HAE-specific medications. However, for a large majority of countries in the world, HAE-specific medications are still not available or accessible. The correspondence on ‘The International/Canadian Hereditary Angioedema Guideline’ tries to address global disparities in HAE care, highlight the key challenges in low- and middle-income countries and provide potentially viable and equitable solution to address disparities.

Hereditary angioedema (HAE) is a rare, potentially life-threatening genetic disorder characterized by recurrent subcutaneous and/or submucosal edema, most commonly caused by deficiency in C1-esterase inhibitor (C1-INH). Patients with HAE also have an impaired quality of life because of the chronic and unpredictable nature of the disease; its implications on productivity and economic burden, relationships as well as impact on the family dynamics [1, 2]. There has been a paradigm shift in treatment goals—from merely managing acute attacks to improving or normalizing quality of life (QoL), thanks to the availability and accessibility of modern, HAE-specific medications [3, 4]. However, for a large majority of countries in the world, HAE-specific medications are still not available or accessible [5]. Moreover, these countries are still struggling with poor diagnostic rates of HAE largely because of lack of awareness, accessibility and/or diagnostic facilities [6]. As a result, the mortality and morbidity because of HAE in these countries remains a major challenge for health care providers [7, 8].

We read with interest the recent ‘The International/Canadian Hereditary Angioedema Guideline’ that provide an evidence-based latest recommendation for managing patients with C1-INH deficiency and normal C1-INH. The first version of these guidelines was published in 2014 with an update in the year 2020. The latest iteration of these international and Canadian guidelines clearly reflects the global paradigm shifts in HAE care and is commendably designed to be ‘applicable to the broadest possible audience.’

The field of HAE has advanced rapidly, with novel on-demand and prophylaxis medications being developed and approved in several developed nations. These updated guidelines help clinicians and HAE specialists to decide the best possible treatment option for their patients based on the latest evidence. The current update of the guidelines also highlights the importance of maintaining a dynamic ‘shared decision making’ between clinicians and patients, while urging payers to respect these collaborative treatment choices, recognizing patient QoL as a key ethical consideration. These guidelines also provide updated recommendations for managing HAE in special populations (e.g. pregnant, lactating mothers and children), offering alternative strategies for countries where plasma-derived C1-INH may not be accessible, and thus serving as a valuable resource for international clinical practice.

Despite the significant advancements in the global landscape of HAE care, as reflected in this guideline update, substantial disparities persist—particularly in resource-limited countries—posing challenges to the effective implementation of these recommendations [5].

One of the key challenges in advancing global HAE care is the limited representation of certain ethnicities and countries in international clinical trials [9]. Data from Asia Pacific regions suggest that of the estimated total number of patients with HAE in 2 most populous countries in the world, i.e., India and China, more than 90% patients remain undiagnosed [10]. Beyond just practical challenges of clinical trial recruitment, the low level of awareness among physicians in these countries remains a key barrier. This contributes to a vicious cycle: limited awareness leads to lower diagnostic rates, which in turn results in poor patient enrolment (Fig. 1). Consequently, governments lack sufficient incentive to approve and support first-line treatment options, and pharmaceutical companies are less motivated to expand their programs into these regions.

**Fig. 1:**
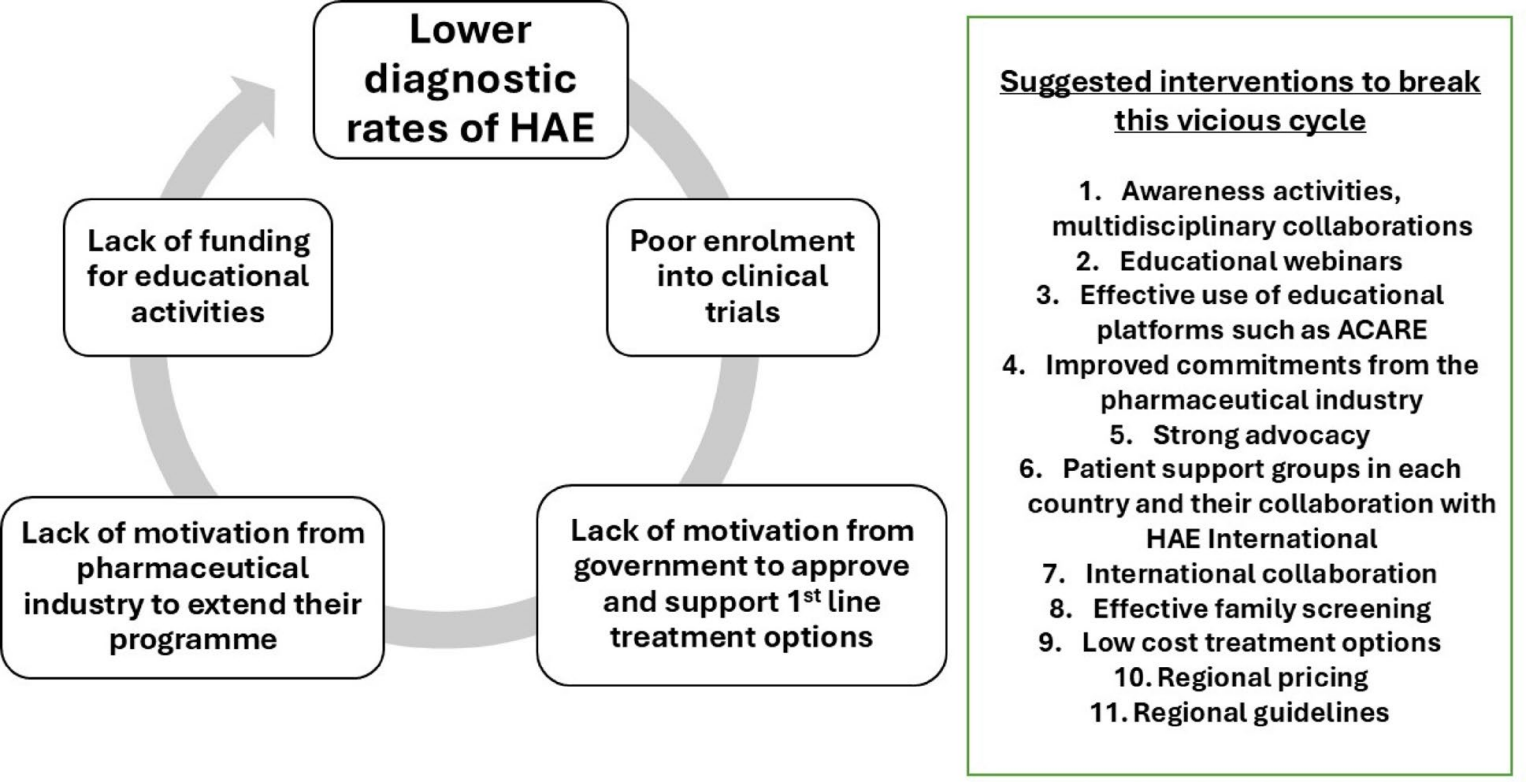
Shows the vicious cycle of events that lead to low diagnostic rates for hereditary angioedema in low-middle income countries and as a result these patients do not get access to the first line treatment options. The figure also enumerates potential solution to break this vicious cycle. Abbreviation: ACARE: Angioedema Centre of Reference and Excellence

The updated guidelines highlight several opportunities to break this vicious cycle, emphasizing the important role that patient support groups—such as HAE International—can play in this effort. As noted in the guidelines, the active involvement of HAE patient support organizations is crucial, having demonstrated significant improvements on patient diagnosis and access [11]. The guidelines also highlight the importance of optimizing diagnostic testing and implementing systematic family screening as key strategies to improve early detection and clinical outcomes in HAE. For example, testing C1-INH levels first, especially in countries where C1-INH function may not be available and combining the use of dried blood spot testing with initiatives such as cascade family screening [12–14].

Even though these guidelines are international, several countries have their own guidelines for management of HAE depending on the availability of the resources in a particular country. In India, for instance, intravenous plasma derived C1-inhibitor concentrate continues to be the drug of choice for on-demand treatment and short-term prophylaxis [14]. Ironically, one of India’s pharmaceutical companies manufactures icatibant, which is marketed globally but not within the country itself. This discrepancy is largely due to the relatively low number of diagnosed HAE patients in India, leading to a perceived lack of market potential. For long-term prophylaxis, however, attenuated androgens and tranexamic acid remain the mainstay of treatment in India.

Another potentially viable and equitable solution to address disparities in treatment access would be to advocate for regional pricing, rather than relying solely on individual country-based cost models. For instance, implementing a more uniform and transparent pricing system across the Asia-Pacific region—based on the projected increase in diagnosed patients once effective treatments become available—could help reduce costs and improve access, as opposed to setting prices based only on the current limited patient numbers in individual countries. Another potential solution could be to develop a low-cost indigenous treatment option. These initiatives would require global collaboration among clinicians, researchers, the pharmaceutical industry, and the international HAE community. However, a critical prerequisite for its success would be the early identification and diagnosis of the large number of currently undiagnosed or “hidden” HAE patients and their families.

We look forward to a future in which international guidelines for the diagnosis and management of HAE can be universally adopted and effectively implemented across all countries—regardless of ethnic, social, racial, or regulatory differences. Achieving this vision will require sustained efforts, strong motivation, and shared commitment from multiple stakeholders, including the global HAE community, patient advocacy organizations, and pharmaceutical partners.

## Data Availability

No datasets were generated or analysed during the current study.
